# Development and characterisation of a panel of phosphatidylinositide 3-kinase – mammalian target of rapamycin inhibitor resistant lung cancer cell lines

**DOI:** 10.1038/s41598-018-19688-1

**Published:** 2018-01-26

**Authors:** Susan Heavey, Paul Dowling, Gillian Moore, Martin P. Barr, Niamh Kelly, Stephen G. Maher, Sinead Cuffe, Stephen P. Finn, Kenneth J. O’Byrne, Kathy Gately

**Affiliations:** 1 Thoracic Oncology Research Group, Trinity Translational Medicine Institute, Trinity College Dublin, Ireland; 2 Biology, NUI Maynooth, Kildare, Ireland; 3 Department of Surgery, Trinity Translational Medicine Institute, Trinity College Dublin, Ireland; 4 Cancer & Ageing Research Program, QUT, Brisbane, QLD Australia

## Abstract

The PI3K-mTOR pathway is involved in regulating all hallmarks of cancer, and is often dysregulated in NSCLC, making it an attractive therapeutic target in this setting. Acquired resistance to PI3K-mTOR inhibition is a major hurdle to overcome in the success of PI3K-mTOR targeted agents. H460, A549, and H1975 resistant cells were generated by prolonged treatment in culture with Apitolisib (GDC-0980), a dual PI3K-mTOR inhibitor over a period of several months, from age-matched parent cells. Resistance was deemed to have developed when a log fold difference in IC50 had been achieved. Resistant cell lines also exhibited resistance to another widely investigated PI3K-mTOR dual inhibitor; Dactolisib (BEZ235). Cell lines were characterised at the level of mRNA (expression array profiling expression of >150 genes), miRNA (expression array profiling of 2100 miRNAs), protein (bottoms-up label-free mass spectrometry) and phosphoprotein (expression array profiling of 84 phospho/total proteins). Key alterations were validated by qPCR and Western blot. H1975 cells were initially most sensitive to Apitolisib (GDC-0980), but developed resistance more quickly than the other cell lines, perhaps due to increased selective pressure from the impressive initial effect. In-depth molecular profiling suggested epithelial-mesenchymal transition (EMT) may play a role in resistance to PI3K-mTOR dual inhibition in NSCLC.

## Introduction

Despite advances in anti-cancer therapies, the overall 5 year survival for lung cancer remains poor, at less than 15%. As such it is crucial that we determine new strategies to overcome this formidable disease. Non-small cell lung cancer (NSCLC) refers to all histological subtypes of lung cancer other than small cell lung cancer, and accounts for ~80% of lung cancers.

Phosphatidylinositol-4,5-bisphosphate 3-kinase (PI3K) signalling can induce all eight hallmarks of cancer in NSCLC and other cancers, and as such a plethora of PI3K targeted inhibitors have been developed in recent years with a view to halting oncogenic signalling in cancer cells^1–5^. Results of early phase clinical trials with single-agent PI3K inhibitors have shown only modest activity in NSCLC with innate and acquired resistance to PI3K pathway inhibition a major hurdle to overcome in the development of these drugs. It is hoped that the mechanisms underlying the development of acquired resistance will highlight potential targetable weaknesses in the resistant tumour phenotype, allowing for the design of a combination approach which reinstates a blockade on survival signalling and allows for a more durable response to treatment.

Acquired resistance to PI3K inhibition has not been well characterised in NSCLC, although mechanisms are beginning to be elucidated in other cancer types. A mouse model engineered to conditionally express *PIK3CA* (H1047R) has revealed that focal amplification of either *MET* or *c-MYC* was present in tumours which reoccurred after *PIK3CA* inactivation. The *ME*T amplified tumours could be inhibited with a selective PI3K inhibitor, but the *c-MYC*-amplified tumours became independent of the PI3K pathway and refractory to treatment with a PI3K inhibitor^6^. *c-MYC* was also independently identified as a candidate PI3K resistance mechanism to dual PI3K-mTOR inhibitor Dactolisib (BEZ235), along with eIF4E^7^. A chemical-genetic screen also revealed *c-MYC* and Notch1 to be involved in resistance to PI3K inhibition^8^. Overexpression of IGF1R was also found to be present in four cell line models of acquired resistance to PI3K inhibition, and IGF1R inhibition was shown to reverse this resistance^9^. AKT3 has also recently been implicated in resistance to the AKT inhibitor, MK2206 in breast cancer^10^.

A growing body of evidence has implicated activation of the epithelial to mesenchymal transition (EMT) program in resistance to targeted therapy^11,12^. EMT is characterized by the upregulation of vimentin expression and inhibition of e-cadherin expression, denoting tissue reprogramming and often associated with a cancer stem cell phenotype. miRNAs are increasingly being implicated in resistance to anti-cancer treatments, including targeting therapies, often through regulation of EMT^13–17^. Furthermore, miRNAs have been shown to be involved in the dysregulation of the PI3K pathway during response/resistance to other treatments, leading us to hypothesize that miRNA may play a role in mediating resistance to PI3K inhibitors, possibly through EMT^18–20^. MiR-205 has been linked to advanced cancers and is a master regulator of EMT. The most prominent gene targets of miR-205 are the e-cadherin transcriptional repressors Zeb1 and Zeb2. Zeb1, Zeb2 and other transcription factors exert their effect by binding to 2 bi-partite E box motifs within the e- cadherin promoter, thereby repressing transcription^11,21–24^.

In this study, three NSCLC cell lines (with different driver mutation profiles) were exposed to the dual PI3K-mTOR inhibitor Apitolisib (GDC-0980) over an extended period with the aim of inducing acquired resistance. Apitolisib was being investigated clinically in NSCLC at the time of the development of these cell lines, though dactolisib (BEZ235) has since become more heavily investigated in the clinical setting^25^. Our apitolisib resistant cell lines have been shown to also exhibit resistance to dactolisib (BEZ235), making these an ideal model for elucidating mechanisms of PI3K-mTOR inhibition. Mechanisms of resistance were characterised at the level of DNA, mRNA, miRNA, protein and protein phosphorylation.

## Materials and Methods

### Cell lines and drugs

H460, A549 and H1975 cell lines were purchased from the European Culture and Tissue Collection. Apitolisib (GDC-0980) was gifted under a material transfer agreement from Genentech for use in this study, and was dissolved in dimethyl sulphoxide (DMSO), aliquoted and stored at −20 °C. Dactolisib (BEZ235) was purchased from Selleckchem, dissolved in DMSO, aliquoted and stored at −20 °C.

### Cell culture

H460 and H1975 cells were grown in RPMI1640 media (Lonza) supplemented with 10% FBS and 1% penicillin/streptomycin at 37 °C and 5% CO_2_. A549 cells were grown in Ham’s F-12 media (Lonza) supplemented with 10% FBS, 1% penicillin/streptomycin and 1% L-glutamine at 37 °C and 5% CO_2_. Three Apitolisib (GDC-0980) resistant cell lines were developed during the course of this study. Lung cancer cell lines H460, A549 and H1975 were treated with IC50 concentrations of Apitolisib (GDC-0980), as calculated from a BrdU proliferation assay for a period of 4–12 months. H460GR (H460 GDC-0980 resistant) cells were maintained in 1.69 µM GDC-0980, A549GR (A549 GDC-0980 resistant) cells were maintained in 3.44 µM GDC-0980 and H1975GR (H1975 GDC-0980 resistant) cells were maintained in 0.58 µM Apitolisib (GDC-0980) in addition to medium supplemented as described above. BrdU assays were carried out each month to assess the development of resistance to Apitolisib (GDC-0980) by comparing resistant cells (H460GR, A549GR and H1975GR) IC50 to age-matched parental cells (H460GP, A549GP and H1975GP) IC50. When matched parent-resistant cell line pairs reached a log fold difference in IC50, acquired resistance was deemed to have developed. All cell lines were tested for mycoplasma once per month by the polymerase chain reaction (PCR) method^26^. Cell line samples from before, during and after the period during which these experiments were carried out were authenticated by DNA Diagnostics Centre.

### Proliferation assays

**Figure 1:**
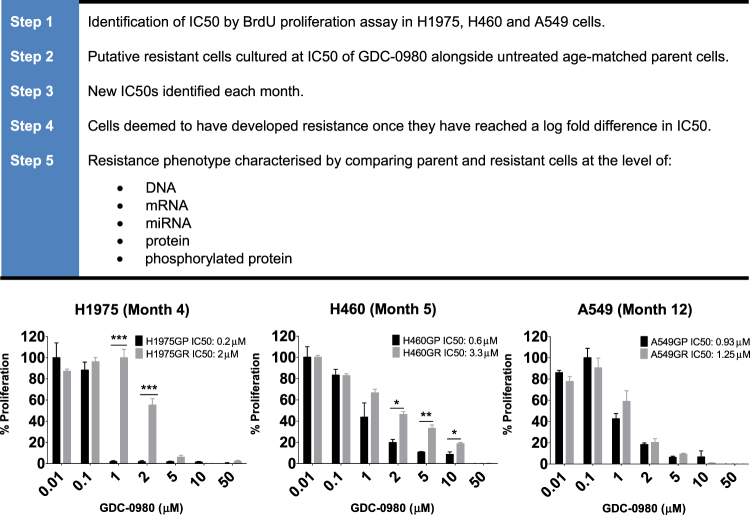
Development of GDC-0980 resistant NSCLC cell lines. A panel of NSCLC cell lines was exposed to GDC-0980 over an extended period in order to develop a cell line model of acquired resistance to the drug. (**a**) Work flow describing the development of GDC-0980 resistant NSCLC cell lines. (**b**) Parent and putative resistant cell lines reached a log fold difference in IC50 at month 4 for H1975 and month 5 for H460. After 12 months, A549 cells did not develop resistance to the drug. Final BrdU proliferation assay results are shown here, where proliferation was normalized on a percentage scale. Assays were performed in triplicate wells and repeated three individual times (n = 3). Data is shown as mean ± SEM. IC50s were calculated separately by linear regression and are noted here. */**/***p < 0.05/0.01/0.001 respectively.

Cell proliferation was measured using a Cell Proliferation enzyme linked immunosorbent assay (ELISA), BrdU (Roche Diagnostics Ltd) and Cell Titre Blue assay (Promega). Cells were seeded at 2000 cells per well in a 96-well plate and adhered overnight. Cells were treated with Apitolisib (GDC-0980) or Dactolisib for 72 hr at a range of concentrations as noted (Fig. 1, Supplementary Figure 1). Following treatment, 10 µL of a 1:1000 dilution of BrdU labelling solution (final concentration-10 µM) was added to each well and plates incubated for 4 hours at 37 °C. Following incubation, the media was removed and the cells fixed and denatured with 200 µL of a fixative solution for 30 minutes at room temperature. 100 µL anti-BrdU-POD (mouse monoclonal antibody, peroxidase-conjugated) working solution was added to each well for 90 minutes at RT. Cells were washed three times with wash buffer and 100 µL of substrate solution was added for 5–10 minutes (or until colour change was sufficient for photometric detection). 25 µL 1 mM H_2_SO_4_ was added to each well to stop the reaction. Absorbance was measured on a Vesamax tunable microplate reader at 450 nm with a reference wavelength set to 690 nm. Alternatively, following treatment, CellTitre-Blue reagent was added to test plate (20 µL per well), shaken for 10 s and incubated for 4hrs, then shaken for 10 s and fluorescence was recorded at 560/590 nm.

### Gene expression arrays

RNA was isolated from parent and resistant cell lines using the RNeasy mini kit (Qiagen). Two RT2 Profiler PCR array panels were used (mTOR signaling and cancer drug resistance) to compare H1975GR with H1975GP and H460GR with H460GP. cDNA was added to RT2 qPCR Master Mix, which contains SYBR Green and reference dye. The experimental cocktail of cDNA, Master Mix and H_2_O was added to the 96 well array (25 μl per well). Real-time PCR thermal cycling was performed using the ABI 7500 thermal cycler. Changes in gene expression between parent and Apitolisib (GDC-0980) resistant cell lines were analyzed using SABiosciences online software which incorporates the ΔΔCT method.

### miRNA expression profiling

miRNA expression profiling was carried out through Exiqon services (Vedbaek, Denmark) in order to identify differentially expressed miRNAs between parent and drug resistant cell lines. RNA was isolated from cell line samples using the miRNeasy kit (Qiagen) and cleaned using the miRCURY RNA isolation kit (Exiqon) as follows. The purified RNA sample was stored at −80 °C and shipped to Exiqon for analysis, where samples were labelled using the miRCURY LNA microRNA Hi-Power Labelling Kit, Hy3/Hy5 and hybridized on the miRCURY LNA microRNA Array (7^th^ generation). Quantified signals were normalized, the background was corrected and supervised and unsupervised data analyses were performed using the Quantile algorithm.

### miRNA validation by RT-PCR

RNA was diluted to 5 ng/μl using RNase-free water. cDNA was synthesised using the miRCURY LNA™ Universal RT microRNA PCR, Starter Kit (Exiqon), briefly 2 μl 5X reaction buffer, 4.5 μl nuclease-free water, 1 μl enzyme mix, 0.5 μl synthetic UniSp6 RNA spike-in and 2 μl template RNA (5 ng/μl). cDNA was amplified using Exilent SYBR Green Master Mix and microRNA LNA™ PCR primer sets U6 snRNA (endogenous control), hsa-miR-205-5p, and UniSp6 (quality control) (Exiqon). Fold change in miRNA expression was calculated using the ∆∆C_t_ method.

### Mass Spectrometry

Cell pellets were lysed in a buffer containing 8 M urea/50 mM NH_4_HCO^3^/0.1% ProteaseMax. The protein amount was estimated using an RC/DC protein assay from Bio-Rad^27^. BSA was used as a standard. After dithiothreitol reduction and iodoacetic acid-mediated alkylation, a double digestion was performed using Lys-C (for 4 hours at 37 °C) and Trypsin (overnight at 37 °C) on 5 µg of protein. Digested samples were desalted prior to analysis using C18 spin columns (Thermo Scientific, UK). 500 ng of digested protein was analysed from each digest using a Q-Exactive mass spectrometer coupled to a Dionex RSLCnano (Thermo Scientific, Waltham, MA, USA). Peptides were separated using a 2% to 40% gradient of acetonitrile (A: 0.1% FA, B: 80% acetonitrile, 0.1% FA) on a Biobasic C18 Picofrit column (ThermoFisher Scientific, Hemel Hempstead, UK) (100 mm length, 75 mm ID) over 65 min at a flow rate of 250 nl/min. Data was acquired with the mass spectrometer operating in automatic data dependent switching mode. A full MS scan at 140,000 resolution and a range of 300–1700 m/z was followed by an MS/MS scan, resolution 17,500 and a range of 200–2000 m/z, selecting the 15 most intense ions prior to MS/MS (Top15 method)^28^.

### Label-free analysis

Progenesis label-free LC-MS software version 3.1 from Non-Linear Dynamics (Newcastle upon Tyne, UK) was used to process the raw data generated from LC-MS/MS analysis. Data alignment was based on the LC retention time of each sample, allowing for any drift in retention time given and adjusted retention time for all runs in the analysis. A reference run was established with the sample run that yielded most features (i.e. peptide ions). The retention times of all of the other runs were aligned to this reference run and peak intensities were then normalized^29^.

Prior to exportation to Proteome Discoverer 1.4 (Thermo Scientific), the MS/MS data files were filtered using the following parameters; (1) peptide features with ANOVA ≤ 0.05 between experimental groups, (2) mass peaks with charge states from +1 to +5 and (3) greater than one isotope per peptide. The PepXML generic file, generated from all exported MS/MS spectra, was used for peptide identification using Proteome Discoverer 1.4 against Sequest HT (SEQUEST HT algorithm, licence Thermo Scientific, registered trademark University of Washington, USA) and searched against the UniProtKB-SwissProt database (taxonomy: Homo sapiens). The following search parameters were used for protein identification: (1) peptide mass tolerance set to 10 ppm, (2) MS/MS mass tolerance set to 0.02 Da, (3) up to two missed cleavages were allowed, (4) carbamidomethylation set as a fixed modification and (5) methionine oxidation set as a variable modification. For re-importation back into Progenesis LC–MS software for further analysis, only peptides with XCorr scores >1.9 (+1), >2.2 (+2) >3.75 (+3) and high peptide confidence were selected. A number of criteria were applied to ensure proper identification/evaluation of cellular proteins, including an ANOVA p-value between experimental groups of ≤0.05, fold change ≥2 and proteins with ≥2 peptides matched^30^.

### Phospho-protein profiling

Human phospho kinase arrays (R&D systems) were used to profile expression of 43 kinase phosphorylation sites and 2 related total proteins across a panel of cell lines. Capture and control antibodies are spotted in duplicate on nitrocellulose membranes. Cell lysates were incubated with these membranes in a multi well dish overnight and detected by chemiluminescence. Proteins were isolated and quantified as per manufacturer’s instructions. Array buffer 1 (block buffer, 1 mL) was added to each well of the dish provided, and array membranes were added to relevant wells and incubated at RT for 1 hour on a shaker. Protein samples were diluted to 500 mg/2 mL with array buffer one, and then added to relevant wells and incubated at 4 °C overnight on a shaker. Three 10 minute washes was performed using 1X wash buffer, then membranes were incubated in detection antibody for 2 hours at room temperature on a shaker. A further three washes were carried out prior to membrane incubation in Strepdavidin-HRP for 30 minutes on a shaker. Three further washes were carried out and then membranes were incubated in Chemi Reagent Mix for 1 minute and spots visualized on a Biospectrum Imaging System. Densitometry analysis was carried out using ImageJ.

PathScan arrays (Cell Signalling Technologies) were used here to profile expression of 28 RTKs and 11 signalling nodes, when phosphorylated at tyrosine or other residues as noted. Protein samples were prepared and quantified as per kit guidelines. 100 µL blocking buffer was added to each well and incubated at RT for 15 minutes on a shaker. Cell lysates are diluted to 1 mg/mL and 75 µL of this working solution is added per well and incubated for overnight at 4 °C on a shaker. Four 5 minute washes were carried out using 1X array wash buffer, and 75 µL detection antibody cocktail was added. The slide was incubated at RT for 1 hour on a shaker, then four further 5 minute washes were carried out. 75 µL HRP-linked Strepdavidin was added to each well and incubated for 30 minutes at RT on a shaker. Four further 5 minute washes were carried out prior to incubation in LumiGlo/peroxide mix (chemiluminescent reagent) for the duration of slide imaging using a Biospectrum Imaging System. Densitometry analysis was carried out using Image J.

### Semi-Quantitative analysis of EMT related genes (Zeb1, Zeb2, E-cadherin) in matched H1975P and H1975GR cell lines

Total RNA from H1975P and H1975GR cell lines was extracted using 1 ml TRI Reagent and miRCURY LNA isolation kit (Exiqon) (as per manufacturer’s protocol). First strand complementary DNA (cDNA) was synthesised using 1 µg of total RNA and Superscript III reverse transcriptase kit (Invitrogen). Zeb1, Zeb2 and E-cadherin were amplified using primer sets outlined as follows: Zeb1 Forward (5′ TTCAAACCCATAGTGGTTGCT 3′), Zeb1 Reverse (5′ TGGGAGACACCAAACCAACTG 3′), Zeb2 Forward (5′ CAAGAGGCGCAAACAAGC 3′), Zeb2 Reverse (5′ GGTTGGCAATACCGTCATCC 3′), E-cadherin Forward (5′ CAGCACGTACACAGCCCTAA 3′), E-cadherin Reverse (5′ GCTGGCTCAAGTCAAAGTCC 3′). All primers sets had annealing temperatures of 60 °C. Amplicons were separated by electrophoresis through a 1% agarose gel and visualized under UV light. Densitometry analysis was carried out using ImageJ.

### Western blot

Total protein was extracted on ice from H1975P and H1975GR cell lines using RIPA lysis buffer to which sodium orthovandate (50 mM), phenylmethylsulfonyl fluoride (PMSF) (100 mM), protease inhibitor cocktail, beta-glycerophosphate (500 nM) and sodium fluoride (500 nM) were added. 30 µg of each protein was separated by electrophoresis through a 12% SDS PAGE gel and proteins were transferred onto methanol activated PVDF membrane. After blocking in 1X TBST buffer containing 5% (w/v) non-fat dry milk for 1 hour at room temperature with gentle agitation, the membrane was washed three times for 5 minutes each in 1X TBST and incubated overnight at 4 °C with gentle agitation in 0.1 µg/mL Vimentin primary antibody (R&D Systems) and blocking buffer (5% (v/v) bovine serum albumin (BSA) in 1X TBST). The membrane was washed three times for 5 minutes each in 1X TBST and incubated for 1 hour at room temperature with gentle agitation in 0.1 µg/mL rabbit anti-goat secondary antibody (Santa Cruz). After washing membrane in 1X TBST bound antibody complexes were detected using the Supersignal West Pico Chemiluminescent Substrate Kit (Thermo Scientific) and exposure to imaging X-Ray film (MG-SR Plus, Konica Minolta). Densitometry was carried out by ImageJ.

### Data availability

All array data will be made available to the public via online repositories.

## Results

H460, A549 and H1975 cells were exposed to Apitolisib (GDC-0980) at IC50 concentrations for 4–12 months. Monthly BrdU proliferation assays were carried out in order to assess the development of acquired resistance to the drug. H1975 (*PIK3CA* and *PIK3R1* mutated) cells, which had been the most sensitive cell line to Apitolisib (GDC-0980), began to show decreased sensitivity to the drug as early as 1 month after commencement of GDC-0980 treatment. The difference in IC50 concentration between parent (H1975GP) and putative resistant (H1975GR) cells reached a log fold at month 4 of Apitolisib (GDC-0980) treatment, at which point the cells were deemed to have developed resistance to the drug (Fig. 1). H460 (*PIK3CA* mutated) cells began to show decreased sensitivity to Apitolisib (GDC-0980) after 2 months of treatment with the drug, and reached a log fold difference in IC50 value between parent (H460GP) and resistant (H460GR) cells at month 5 of treatment. At this point the cells were deemed to have developed resistance to the drug (Fig. 1). A549 (*PIK3CA* wild-type) cells had been the least sensitive to PI3K treatment, with the highest IC50 concentration of the three cell lines examined. This cell line did not develop resistance to the drug after 12 months of treatment (Fig. 1). H1975GR cells were noted to also exhibit resistance to Dactolisib (BEZ235), a commonly investigated PI3K-mTOR dual inhibitor (Supplementary Figure 1).

**Figure 2:**
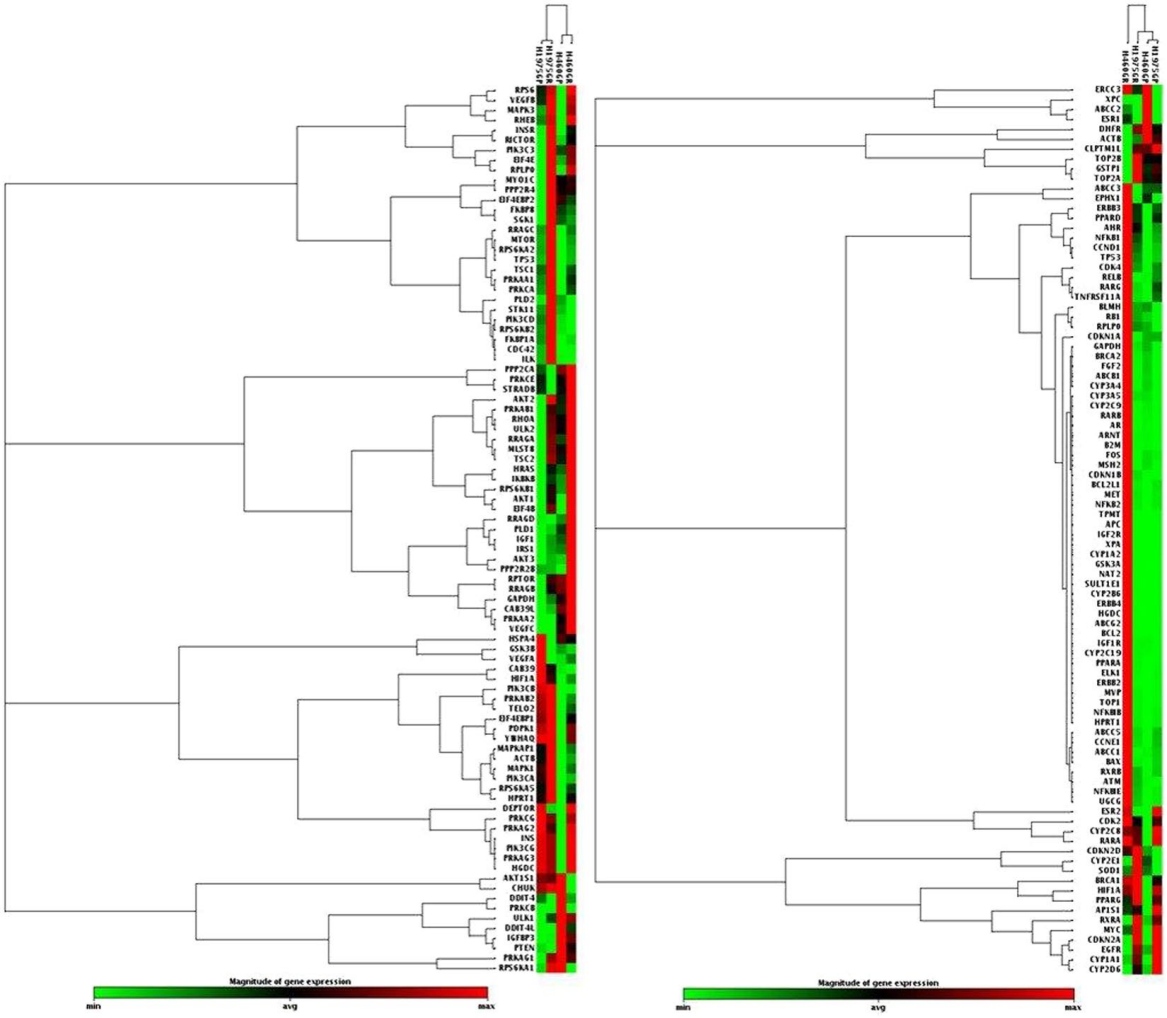
mRNA profile of GDC-0980 resistant NSCLC cell lines. Gene expression was assessed using RT^2^ profiler arrays from SABiosciences (left: mTOR panel, right: cancer drug resistance panel) in order to compare gene expression in H1975GP, H1975GR, H406GP and H460GR cell lines. A clustergram was constructed using SABiosciences online software.

**Figure 3:**
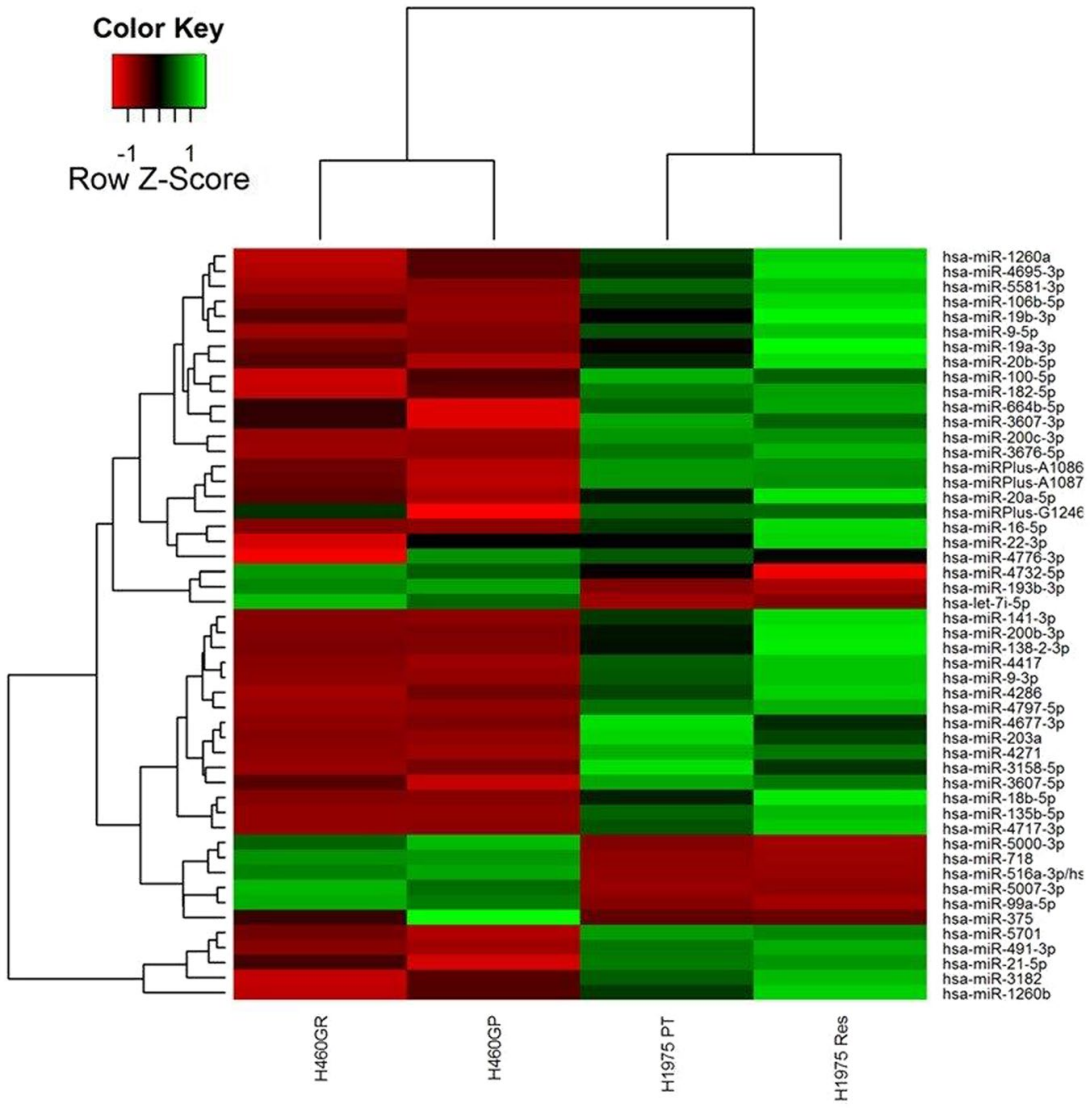
Heat map and hierarchical clustering of H460GP, H460GR, H1975GP and H1975GR miRNA screen. miRNA have previously been shown to regulate resistance to targeted therapies. Here, H460GP, H460GR, H1975GP and H1975GR miRNA samples were screened for miRNA expression by Exiqon services using the miRCURY LNA microRNA array. The top 50 miRNAs (in terms of differential expression) are hierarchically clustered here.

Having developed two cell line models of resistance to Apitolisib (GDC-0980) (H1975GR and H460GR), an in-depth characterisation of the mechanisms of resistance to the drug was carried out. Data presented at the American Association for Cancer Research Special Conference: Targeting the PI3K-mTOR Network in Cancer showed that none of the resistant cell lines exhibited altered mutational profiles compared with matched parent cells^31^. Apitolisib (GDC-0980) resistant cell lines were compared with age matched parental controls using mRNA expression pfrofile arrays (SABiosciences). Two array panels were used here: one which profiles expression of 84 genes related to cancer drug resistance, and one which profiles expression of 84 genes related to mTOR pathway signalling. H1975GR cells were found to overexpress aryl hydrocarbon receptor nuclear translocator (*ARNT*) 53.45 fold, and have a downregulation of estrogen receptor beta (*ESR2*) 53.76 fold (Fig. 2), relative to parental cells. H1975GR cells were also found to overexpress *AKT3* (174.7 fold) and insulin receptor (*INSR*) (5.56 fold), In addition to overexpression of *PIK3C3* (3.59 fold), *PIK3CD* (3.28 fold), *MTOR* (2.18 fold) and a downregulation of DEP domain-containing mTOR-interacting protein (*DEPTOR)* 4.73 fold (Fig. 3). H460GR cells were also found to overexpress *ARNT* (6.32 fold), *ERBB2* (15.97 fold), *ERBB3* (12.35 fold) and *ERBB4* (18329.77 old), while *EGFR* (−314.11 fold) and *MYC* (−4.89 fold) were downregulated (Fig. 2). H460GR cells showed overexpression of *AKT3* (9.26 fold) and a downregulation of *INSR* (2.44 fold), and *PRKCB* (129.40 fold) (Fig. 2).

MicroRNA expression profiling was carried out in H460GP, H460GR, H1975GP and H1975GR samples, using 7th generation miRCURY LNA microRNA Arrays. The total number of miRNAs which were expressed above background was 489. The top 50 of these (by magnitude of difference) are included in a heat map in Fig. 3. The most highly upregulated miRNA in H460GR cells compared to their parental H460GP counterparts was hsa-miRPlus-G1246-3p, while the most downregulated was hsa-miR-375. Hsa-miR-130a-3p was the most upregulated miRNA in H1975GR cells compared with H1975GP cells, and the most downregulated was hsa-miR205-5p (Fig. 3). Validation of EMT regulator hsa-miR205-5p by qPCR confirmed its expression in H1975GP cells however it was undetectable in H1975GR cells.

**Figure 4:**
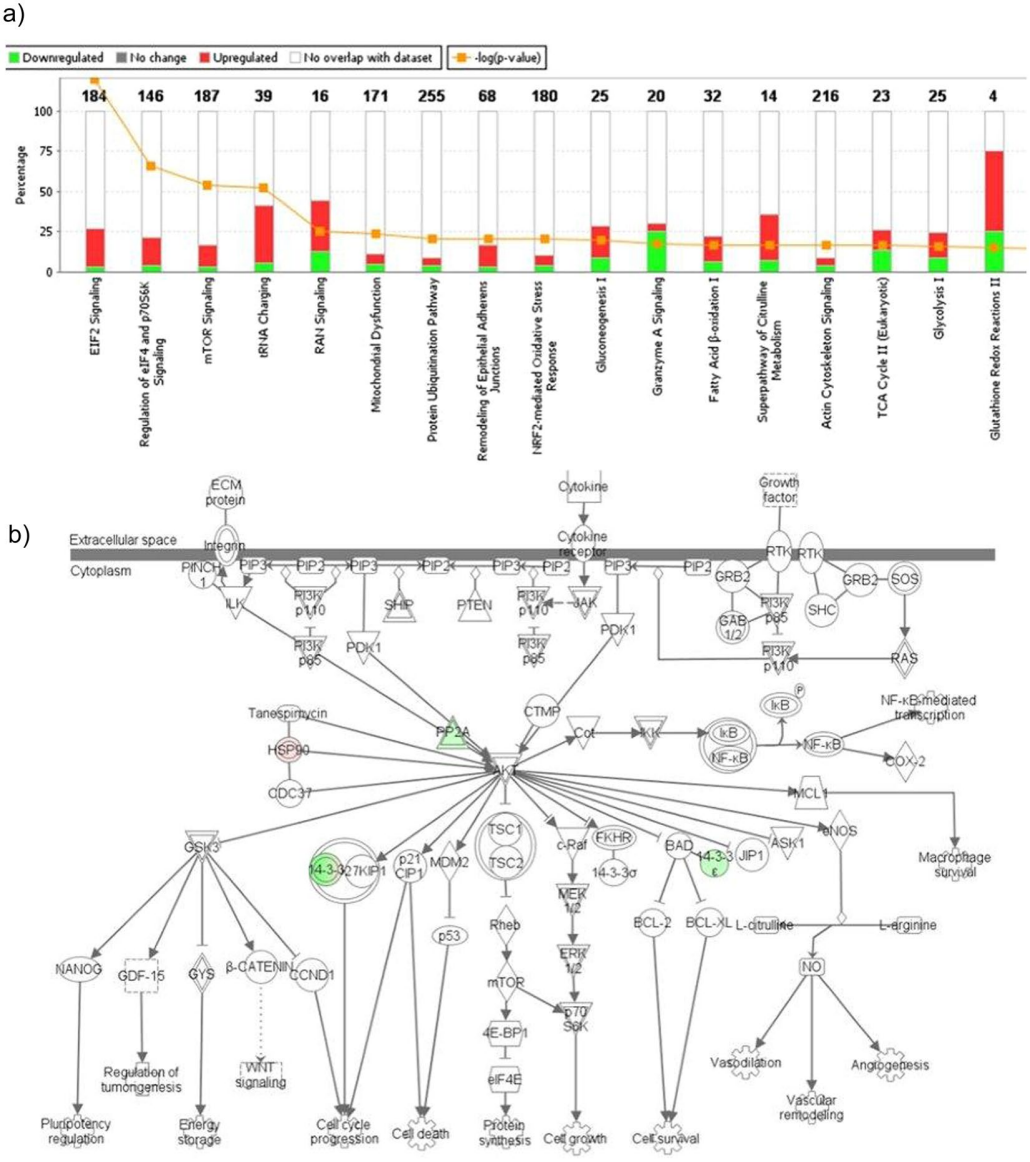
Proteomic analysis of H460GP and H460GR cell lines. Protein was isolated from H460GP and H460GR cell lines and analysed by bottoms-up label-free mass spectrometry, in order to identify differences in protein abundance (n = 3). 592 proteins were significantly (p < 0.05) differentially regulated (fold change >2) between parent and GDC-0980 resistant cells. Data was analysed using Ingenuity Pathway Analysis. (**a**) Top dysregulated pathways are shown. (**b**) Differential regulation is shown in the context of the PI3K pathway.

**Figure 5:**
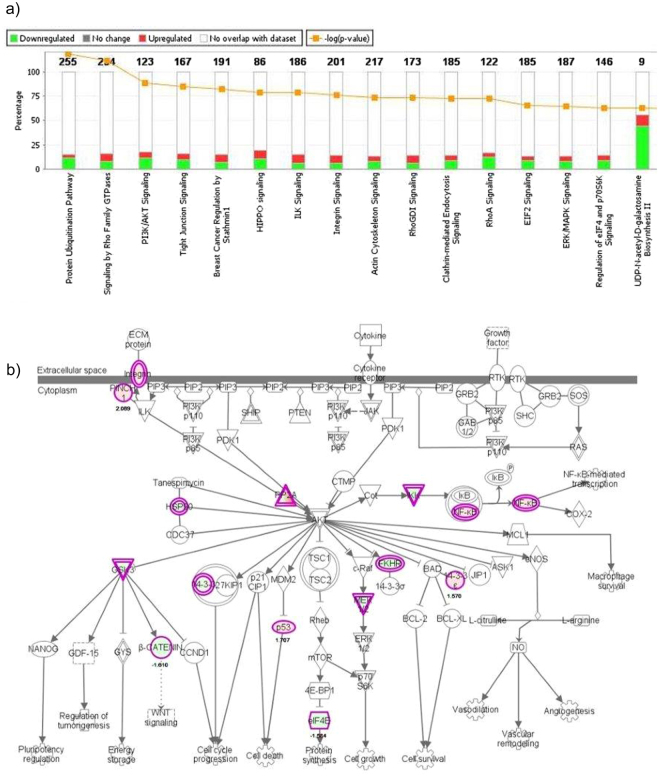
Proteomic analysis of H1975GP and H1975GR cell lines. Protein was isolated from H1975GP and H1975GR cell lines and analysed by bottoms-up label-free mass spectrometry, in order to identify differences in protein abundance (n = 3). 1173 proteins were significantly (p < 0.05) differentially regulated (fold change >2) between parent and GDC-0980 resistant cells. Data was analysed using Ingenuity Pathway Analysis. (**a**) Top dysregulated pathways are shown. (**b**) Differential regulation is shown in the context of the PI3K pathway.

Proteomic analysis of H460GP, H460GR, H1975GP and H1975GR cell lines was carried out using bottoms-up label-free mass spectrometry (n = 3). 592 proteins were >2 fold differentially regulated, with p < 0.05 between H460GP and H460GR cell lines (Fig. 4). KEGG analysis highlighted activation of several regulators of EMT including vimentin (13 fold), desmin (12 fold) and filamin (28 fold) in H1975GR cell lines. The dataset was analysed using Ingenuity Pathway Analysis software, which identified alterations in eif2, eif4 and mTOR signalling. 1173 proteins were >2 fold differentially regulated, with p < 0.05 between H1975GP and H1975GR cell lines. Alterations in pathways identified between these two cell lines included ubiquitination, Rho and PI3K/AKT signalling (Fig. 5).

Proteomic analysis of H1975GP and H1975GR cells highlighted a large number of proteins that may be involved in resistance to Apitolisib (GDC-0980). This was further investigated by interrogating intracellular signalling pathway activation by phosphorylation. To achieve this, both H1975GR and H460GR cell lines were compared with their age-matched parental control cell lines using phospho-kinase arrays. (Supplemental Figure 2). H1975GR cells exhibited increased expression of AKT1/2/3 (T308) (2.03 fold), and decreased expression of PRAS40 (T246) (33.85 fold), AKT1/2/3 (S473) (15.29 fold), among others. H460GR cell exhibited increased expression of EGFR (Y1086) (1.47 fold), AKT1/2/3 (S473) (3.3 fold), ERK1/2 (T202/Y204) (2.8 fold) and p38α (T180/Y182) (8.2 fold) and decreased expression of p53 (S392, S46 and S15) (1.66 fold, 4.64 fold and 2.54 fold respectively), among others.

Further analysis of H1975GR cells using Ingenuity Pathway Analysis identified a number of alterations in proteins involved in epithelial-mesenchymal transition (EMT).

**Figure 6:**
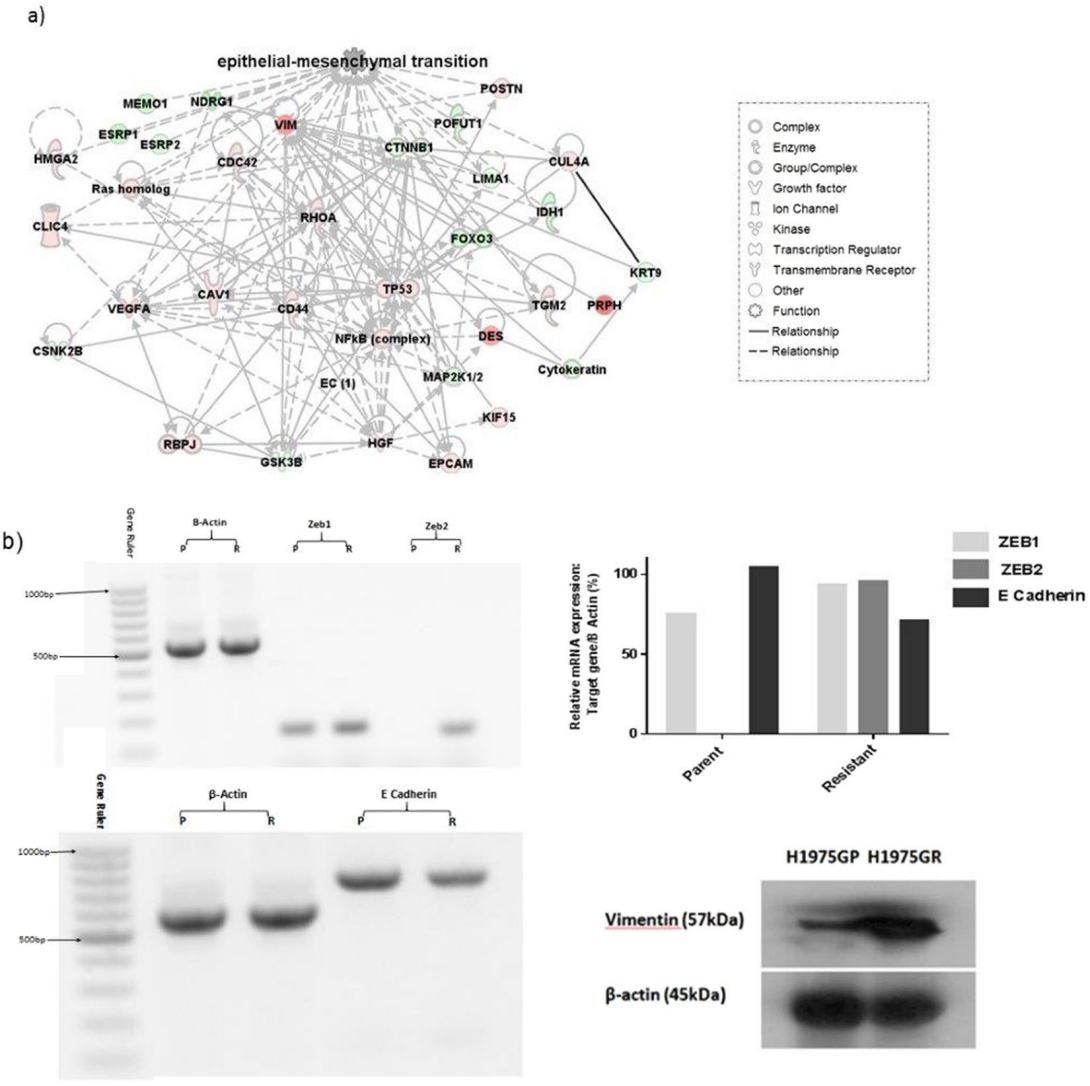
Dysregulation of EMT in GDC-0980 resistant cells. (**a**)H1975PT and H1975GR cells were analysed by bottoms-up label-free mass spectrometry in Fig. 4. Ingenuity pathway analysis revealed significant (p < 0.05) dysregulation in a number of proteins involved in EMT, (red = upregulation, green = downregulation). (**b**)PCR and Western blotting was carried out in order to validate the EMT dysregulation identified by mass spectrometry in part a. PCR data showed downregulation of E-cadherin and upregulation of Zeb1 & Zeb2 mRNA expression in H1975GR vs H1975GP. Western blot analysis showed elevated vimentin expression.

Figure 6 downregulation of E-cadherin and upregulation of ZEB1 and ZEB2 were confirmed at the mRNA level by PCR, while upregulation of Vimentin protein was confirmed by Western blot (Fig. 6).

## Discussion

This study set out to develop NSCLC cell line models of resistance to Apitolisib (GDC-0980), a dual PI3K-mTOR inhibitor which is currently in Phase II clinical trials for lymphomas and solid tumours. H1975GR cells were noted to also exhibit resistance to Dactolisib (BEZ235), another commonly investigated PI3K-mTOR dual inhibitor in Phase II trials for cancer. The cell line models were characterised in detail with a view to identifying targetable mediators of resistance to the drug.

H460, A549 and H1975 cells were exposed to IC50 concentrations of Apitolisib (GDC-0980) over an extended period of time in order to develop cell line models of acquired resistance to the drug. H1975 cells, which were the most sensitive cell line to Apitolisib (GDC-0980) treatment, were the first to develop resistance. In fact, this cell line began to exhibit decreased sensitivity to the drug after just one month, and developed a log fold difference in IC50 concentration between parent (H1975GP) and resistant (H1975GR) cell lines after just 4 months of treatment with Apitolisib (GDC-0980). H1975 cells were shown to harbour mutations in both *PIK3CA* and *PIK3R1*, and have previously been shown to express PI3K pathway signalling phosphoproteins more highly than the other cell lines used here^32^.

It is hypothesized that the initial sensitivity to PI3K inhibition here could imply a reliance on PI3K pathway signalling, with the cells being addicted to the pathway. As such, the drug mediated significant effects in the short term, but the increased selective pressure lead to the cells becoming rapidly resistant to the drug.

H460 cells, which were also sensitive to Apitolisib (GDC-0980), though not as sensitive as H1975 cells, were the second cell line to develop drug resistance. Here, an initial decrease in sensitivity to Apitolisib (GDC-0980) was observed after 2 months, and a log fold difference in IC50 achieved after 5 months of treatment. H460 cells were shown to harbour a mutation in *PIK3CA* but not *PIK3R1*, and to express PI3K signalling molecules at lower levels than H1975 cells. This cell line, unlike H1975 cells, also harbours a mutation in *KRAS*. As such it is hypothesized that this cell line does utilise PI3K signalling, but is not addicted to the pathway. Previously it has been hypothesized that some tumours could be dependent on mutant *PIK3CA* as a driver oncogene, whereas in other cases, the *PIK3CA* mutation may modulate the effect of another oncogenic process^33^. We hypothesize that H1975 cells represent an example of the former, and H460 cells represent an example of the latter, where mutant *KRAS* is the driver mutation. As such, while H460 cells were less sensitive to PI3K inhibition initially, the effects of the drug were sustained over a longer period due to the reduced selective pressure.

Based on these data, we would hypothesize that patients who exhibit PI3K pathway activation, but not oncogene addiction to mutant *PIK3CA*, will undergo a moderate response to PI3K-mTOR inhibition, which will be sustained over a longer period than patients whose tumours exhibit addiction to mutant *PIK3CA*.

A549 cells were previously shown to exhibit mutated *KRAS* but wild-type *PIK3CA*, and were least sensitive to Apitolisib (GDC-0980), initially having a higher IC50 concentration than the other three cell lines. This cell line does not appear to rely on PI3K signalling, and may exhibit low levels of innate resistance to Apitolisib (GDC-0980), although there is no established cut-off to define whether cells exhibit true innate resistance, or merely reduced sensitivity to the compound. After 12 months of treatment with Apitolisib (GDC-0980), A549 cells had not developed further resistance to the drug. As such, patients who do not exhibit significant activation of the PI3K pathway may benefit, in part, from PI3K inhibition, in that it may induce minimal effects, but sustain these effects over a longer period.

Detailed molecular characterisation of H1975GR and H460GR cell lines was carried out relative to their matched parent cell lines, at the level of DNA, mRNA, total and phospho-proteins. Several key trends were observed. *AKT3* gene expression was greatly increased in all three Apitolisib (GDC-0980) resistant cell lines compared to their matched parent cell lines. *AKT3* is the least studied isoform of AKT, with its precise role in cell signalling being poorly understood. Nonetheless, the gene has been associated with multiple disease phenotypes, mostly including neurological developmental defects due to its known role in brain development^34^. In relation to cancer, *AKT3* has been implicated in the development of glioblastoma multiforme^35^, malignant melanoma^36^ and may contribute to a more aggressive clinical phenotype in estrogen receptor-negative breast cancers and androgen-insensitive prostate carcinomas^37^. Furthermore, *AKT3* may contribute to cisplatin resistance in human uterine cancer cells^38^. Increased expression of *AKT3* was associated with a decrease in expression of *ERS2* in H1975GR cells, and a decrease in expression of *ESR1* in H460GR cells. Correspondingly, in a study by Grabinski *et al*. (32), inactivation of *AKT3* was shown to result in increased expression of ERα. *AKT3* was also shown to regulate *ERBB2* and *ERBB3*, which are both upregulated in H460GR cells. Recently, knockdown of *AKT3* in conjunction with *PIK3CA* has been shown to suppress cell viability and proliferation and induce apoptosis of glioblastoma multiforme cells^39^, and *AKT3* has been implicated in resistance to the AKT inhibitor, MK2206^40^. With increasing interest in a role for *AKT3* in cancer, there may be a future role for *AKT3* targeted therapies, which we hypothesize may be useful in the setting of PI3K-mTOR inhibitor resistance.

Based on previous work in acquired resistance to PI3K inhibition^9^, the IGF-1 pathway was anticipated to play a role in acquired resistance to Apitolisib (GDC-0980) here. While there was some dysregulation of the pathway observed in H1975GR cells by mass spectrometry, there was nothing to suggest a categorical shift to IGF1 signalling. Again in H460GR cells, there was some dysregulation of IGF related genes observed at the level of mRNA, but nothing to suggest a significant shift in signalling to this pathway. H460GR cells displayed a marked switch from *EGFR* expression to *ERBB2, ERBB3* and *ERBB4* expression which may imply a targetable bypass mechanism of resistance is underway in these cells. Based on the known level of cross talk between the PI3K and MAPK pathways and previously published synergistic interaction between PI3K and MEK inhibitors in NSCLC^32^, it was also anticipated that MAPK signalling may play a role in Apitolisib (GDC-0980) resistance. While some MAPK family proteins were differentially regulated in H1975 cells, as observed by mass spectrometry, there was no evidence of a categorical shift in signalling.

Growing data underpins the importance of EMT in lung cancer, with cells that take on a more mesenchymal phenotype becoming more motile, allowing for increased aggression and therefore tumour progression. A recent study found that EMT marker expression in the leading edge of NSCLC tumours correlates with advanced stage and poor differentiation^41^, with other recent studies highlighting the association between EMT and proliferation and invasion^42^, metastasis^43^ and poor prognosis^44^ in NSCLC. A growing body of evidence supports a role for EMT in resistance to targeted therapies in NSCLC and other cancers^45^, with the EMT phenotype seemingly allowing cells to overcome drug inhibition. Resistance to sorafenib, a tyrosine kinase inhibitor that targets proteins such as VEGF, PDGFR and the Raf family, has recently been shown to be mediated by EMT using xenografts from an A549 cell line model of resistance^46^. An A549 cell line model of resistance to gefitinib (an EGFR inhibitor) has also been recently published, again with EMT identified as a potential mechanism of resistance due to altered EMT marker expression^47^. Here, proteomics analysis revealed a dysregulation in pathways related to EMT in H1975GR cells. miR205-5p, was noted to be overexpressed in H1975GR cells, which has been shown to target genes that regulate EMT, and is associated with cancer progression^48^. Further investigations confirmed dysregulation of well-known EMT markers E-cadherin, vimentin, Zeb1 and Zeb 2 in this cell line, supporting this aggressive EMT phenotype in H1975GR cells. H1975GR cells were shown to overexpress miR-1260b and miR-19a-3p, which have been linked with lymph node metastasis in NSCLC and colorectal cancer respectively, supporting the aggressive phenotype of these cells^49,50^.

These data strengthen the evidence base for the roles of EMT, AKT3 and the ERBB family in targeted therapy resistance. These cell line models of resistance, along with the molecular characterisation datasets made available here, will provide a valuable resource to study targeted therapy resistance moving forward, particularly as the cell lines are resistant to both Apitolisib (GDC-0980) and Dactolisib (BEZ235).

## Electronic supplementary material

Supplementary datatsets
