# Supplementary material for: Development and characterisation of a panel of phosphatidylinositide 3-kinase – mammalian target of rapamycin inhibitor resistant lung cancer cell lines
Source: Sci Rep. 2018 Jan 26;8:1652. doi: 10.1038/s41598-018-19688-1 (PMC5786033; doi:10.1038/s41598-018-19688-1)
Supplement: Supplementary file 1 — Supplementary datatsets [file 41598_2018_19688_MOESM1_ESM.doc]

**Title:** Development and characterisation of a panel of phosphatidylinositide 3-kinase – mammalian target of rapamycin inhibitor resistant lung cancer cell lines

**Authors and affiliations:** Susan Heavey^1^, Paul Dowling^2^, Gillian Moore^1^, Martin P. Barr^1^, Niamh Kelly^1^, Stephen G. Maher,^3^ Sinead Cuffe^1^, Stephen P. Finn^1^, Kenneth J. O'Byrne^4^, Kathy Gately^1^ .

1 Thoracic Oncology Research Group, Trinity College Dublin/St. James's Hospital, Dublin, Ireland

2 Biology, NUI Maynooth, Kildare, Ireland

3 School of Biological, Biomedical and Environmental Science, University of Hull, Hull, UK

4 Cancer & Ageing Research Program, QUT, Brisbane, QLD, Australia

**Running Title:** Development and Characterisation of PI3K-mTOR resistant NSCLC cells

**Keywords:** PI3K, mTOR, NSCLC, lung, resistance

**Grant support:** Irish Cancer Society, CRS11HEA

**Corresponding author:** Susan Heavey

Address: Thoracic Oncology Research Group, Trinity College Dublin/St. James's Hospital, Dublin, Ireland

Email: [heaveys@tcd.ie](mailto:heaveys@tcd.ie)

Phone: 07427068859

Fax: N/A

**Conflict of Interest:** The authors have no conflicts of interest to declare

**Supplementary data file.**

**Supplementary Figure 1: H1975 cells resistant to Apitolisib (GDC-0980) are also resistant to Dactolisib (BEZ-235)**

H1975GP and H1975GR cells were treated with increasing doses of PI3K-mTOR dual inhibitor Dactolisib (BEZ-235) for 72hr and cell viability was assessed by cell titerre blue assay (n=3). Data is shown as mean ±SEM and IC50s were calculated by non-linear regression.


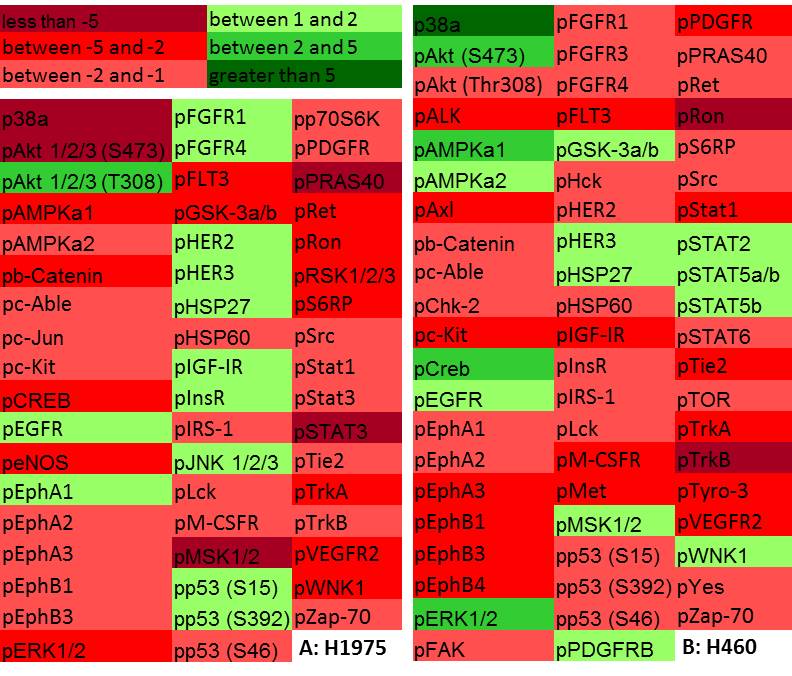


**Supplementary Figure 2: phospho protein profile of GDC-0980 resistant NSCLC cell lines**

Protein was isolated from GDC-0980 resistant cell lines and their age matched parental controls and incubated with phospho kinase arrays (R&D) or PathScan arrays (CST) overnight, then developed as per manufacturer’s protocol. Array images were analysed by densitometry using ImageJ, and fold changes were calculated for H1975GR cells compared to H1975GP cells (left) and H460GR cells compared to H460GP cells (right). Green = upregulation, red = downregulation.

**Supplementary Gel images, related to Figure 6:**

**Full Beta actin: Full Vimentin:**

**
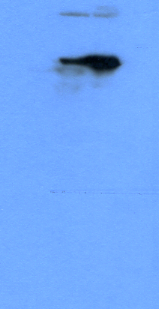

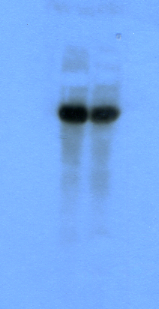
**


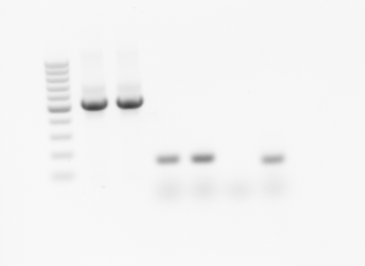
**Full Zeb 1/2:**


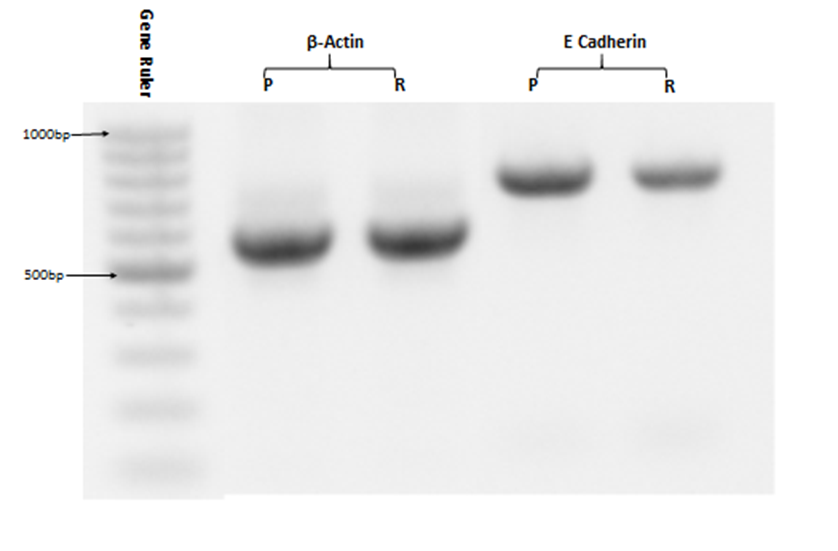
**Full E Cadherin / Beta Actin:**

**
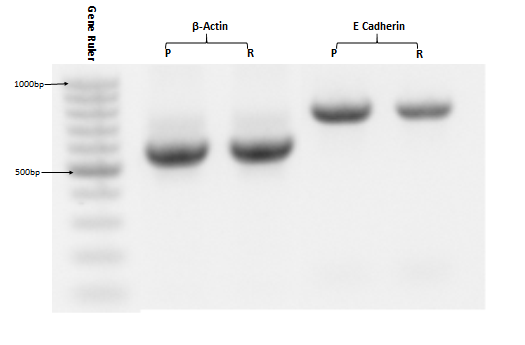
**
